# McGill University

**Published:** 1895-05

**Authors:** 


### McGILL UNIVERSITY.

The commencement exercises of the veterinary department of the McGill University were held on Friday, March 29th, in the William Molson Hall, and were presided over by Chancellor Sir Donald Smith. Seated with him on the platform were Messrs. J. H. R. Molson, W. C. McDonald, S. Finley, C. J. Fleet, Dr. Alex. Johnson, vice-principal; Sir William Dawson, Dr. D. McEachran, dean of the faculty; Dr. Girwood, Dr. Charles McEachran, Prof. Adami, Prof. Penhallow, Prof. Bovey, Rev. D. Clark Murray, Dr. W. S. Morrow, Mr. J. W. Brakenridge, acting secretary; Dr. Gadsden, Philadelphia; Dr. John M. Parker, Haverhill, Mass., and Dr. Miller, Burlington, Vt., the examining committee of the graduating class.

After prayer by Rev. Dr. Clark Murray, Dean McEachran read the report of the college work, after which the following prizes and scholarships were awarded the successful competitors: Veterinary medicine and surgery—W. K. Inglis. Anatomy—Harri Dell. Cattle pathology—C. H. Zink. Cynology—C. H. Zink. Botany—F. A. Brennan. Chemistry—C. H. Higgins. Physiology—Harri Dell. Pharmacology and therapeutics—C. H. Higgins. Best general examination in all subjects (silver medal)—John Campbell Hargrave.

Scholarships: For the highest aggregate obtained in first year subjects ($50)—F. A. Brennan. For the highest aggregate obtained in second year subjects ($50)—H. Dell.

The presentation of diplomas was then proceeded with, the oath being administered by Dr. Alex. Johnson, who also performed the "capping," the acting secretary handing the diplomas to the following-named graduates: W. K. Inglis, Granby, Quebec; J. C. Hargrave, Medicine Hat; W. V. Jones, Wolfville, N. S.; C. H. Zink, Jr., Philadelphia; H. D. Clark, Plainfield, Mass.; L. S. Cleaves, Boston, Mass.; E. H. Lehnert, Clinton, Mass.; A. Cowan, Montreal; C. A. Boutelle, Danville, Quebec; J. C. Cutting, Boston, Mass.; W. A. Morrin, Belle Riviere, Quebec.

The valedictory address was delivered by C. H. Zink, of Philadelphia, who spoke warm words on behalf of his alma mater. The address of Dean McEachran, as usual, was replete with wholesome advice, a synopsis of which appears in this number of the JOURNAL.

The graduates were afterward addressed by Sir Donald Smith, who urged them to honorable work on behalf of the college and in the interests of the great agricultural industry of Canada.

Sir William Dawson in his remarks showed the close relationship of veterinary science and agriculture, and said that it was the duty of everyone connected with the veterinary profession to aid in improving live stock as well as caring for them in disease, as the improvement of live stock was one of great importance to the community. He considered that it was the duty of every educated man in the country to make himself an expert in scientific agriculture, and thus to promote and extend its welfare. Further, that good stock could not be kept unless there was good food to feed them on, and their proper care made a thorough study, closing with the remark that it was not by speculating in the stock market that the country was to be built up; it was by improving the animals.

Dr. Alex. Johnson expressed the hope and wish that the term required for study would soon be extended, and that veterinary science would still take higher rank.

The proceedings closed with the benediction pronounced by Rev. Dr. Clark Murray.

